# Discrepant Prevalence and Incidence of *Leishmania* Infection between Two Neighboring Villages in Central Mali Based on Leishmanin Skin Test Surveys

**DOI:** 10.1371/journal.pntd.0000565

**Published:** 2009-12-15

**Authors:** Fabiano Oliveira, Seydou Doumbia, Jennifer M. Anderson, Ousmane Faye, Souleymane S. Diarra, Pierre Traoré, Moumine Cisse, Guimba Camara, Koureissi Tall, Cheick A. Coulibaly, Sibiry Samake, Ibrahim Sissoko, Bourama Traoré, Daouda Diallo, Somita Keita, Rick M. Fairhurst, Jesus G. Valenzuela, Shaden Kamhawi

**Affiliations:** 1 Laboratory of Malaria and Vector Research, National Institute of Allergy and Infectious Diseases, National Institutes of Health, Rockville, Maryland, United States of America; 2 Faculty of Medicine, Pharmacy and Odontostomatology, University of Bamako, Bamako, Mali; 3 Centre National d'Appui à la Lutte contre la Maladie, Bamako, Mali; Institut Pasteur de Tunis, Tunisia

## Abstract

Apart from a single report, the last publication of cutaneous leishmaniasis (CL) in Mali dates back more than 20 years. The absence of information on the current status of CL in Mali led us to conduct a cohort study in Kemena and Sougoula, two villages in Central Mali from which cases of CL have been recently diagnosed by Mali's reference dermatology center in Bamako. In May 2006, we determined the baseline prevalence of *Leishmania* infection in the two villages using the leishmanin skin test (LST). LST-negative individuals were then re-tested over two consecutive years to estimate the annual incidence of *Leishmania* infection. The prevalence of *Leishmania* infection was significantly higher in Kemena than in Sougoula (45.4% vs. 19.9%; OR: 3.36, CI: 2.66–4.18). The annual incidence of *Leishmania* infection was also significantly higher in Kemena (18.5% and 17% for 2007 and 2008, respectively) than in Sougoula (5.7% for both years). These data demonstrate that the risk of *Leishmania* infection was stable in both villages and confirm the initial observation of a significantly higher risk of infection in Kemena (OR: 3.78; CI: 2.45–6.18 in 2007; and OR: 3.36; CI: 1.95–5.8 in 2008; P<0.005). The absence of spatial clustering of LST-positive individuals in both villages indicated that transmission may be occurring anywhere within the villages. Although Kemena and Sougoula are only 5 km apart and share epidemiologic characteristics such as stable transmission and random distribution of LST-positive individuals, they differ markedly in the prevalence and annual incidence of *Leishmania* infection. Here we establish ongoing transmission of *Leishmania* in Kemena and Sougoula, Central Mali, and are currently investigating the underlying factors that may be responsible for the discrepant infection rates we observed between them.

**Trial Registration:**

ClinicalTrials.gov NCT00344084

## Author Summary

Leishmaniasis is a vector-borne disease transmitted to humans by the bite of an infected sand fly. Leishmaniasis is present in more than 88 countries and affects more than 12 million people. Depending on the species of *Leishmania*, the host can develop cutaneous leishmaniasis (CL), which is characterized by skin ulcers in uncovered parts of the body or a more severe form, visceral leishmaniasis, which affects the liver and spleen and is fatal if not treated. This study aims to establish the past and present infection with *Leishmania* parasites in two villages where recent cases have been diagnosed by the dermatology center (CNAM) in Bamako. This was achieved using a *Leishmania*-specific skin test that was administered annually to permanent residents of Kemena and Sougoula villages from 2006 to 2008. The results show that transmission of *Leishmania* is active and stable in these two villages. Moreover, despite sharing similar cultural and environmental features, the individuals from Kemena presented three times the risk of *Leishmania* infection compared with those from Sougoula. Our findings raise awareness of the continued presence of CL in Mali.

## Introduction

Leishmaniasis is endemic in 88 countries with an estimated global burden of approximately two million disability-adjusted life years [1]. No vaccine is currently available for leishmaniasis, and chemotherapy—if accessible—is costly and has considerable side effects [2],[3]. Leishmaniasis is a vector-borne disease transmitted to the host via the bite of a *Leishmania*-infected phlebotomine sand fly. Depending on the *Leishmania* species, humans can develop visceral or cutaneous forms of the disease. In the Old World, cutaneous leishmaniasis (CL) is a self-healing disease characterized by skin lesions that can develop into unsightly scars, typically on the face and extremities. CL was first reported from Mali, West Africa, in 1958 [4]. Since then there have been sporadic reports of CL in Mali [5]–[13], with the majority of cases originating from 5 of 42 districts (Kayes, Bafoulabe, Segou, Gao, and Nara) [6]. *Leishmania major* was first identified as the causative agent of CL in Mali following its isolation from two skin lesions, one from a tourist and the other from a permanent resident of Mali [10],[11]. Both individuals were likely infected in the Mopti region of Central Mali. The parasite isolates were characterized by isoenzyme analysis as *L. major* MON-26 and MON-25 [10],[11]. Recently, two additional zymodemes of *L. major*, MON-74 and MON-117, were reported from Mali[13]. Keita et al. [12] reported on CL cases referred to the Centre National d'Appui à la Lutte contre la Maladie (CNAM), the only dermatological reference center in Mali, from 1997 to 2001, showing that CL remains widely distributed in the country. Thirty of the 251 CL cases (12%) reported in this study originated from the Segou district of Mali [12].The present study demonstrates the active transmission of *Leishmania* infection in two neighboring villages (Kemena and Sougoula) in the Segou district of Central Mali.

## Methods

### Ethics statement

The study protocol (NCT00344084, available at http://www.clinicaltrial.gov/) was approved by the Institutional Review Boards of the National Institute of Allergy and Infectious Diseases (USA) and the University of Bamako (Mali). The study was externally monitored for protocol agreement, data integrity, and protection of human subjects.

### Study area

**Figure 1:**
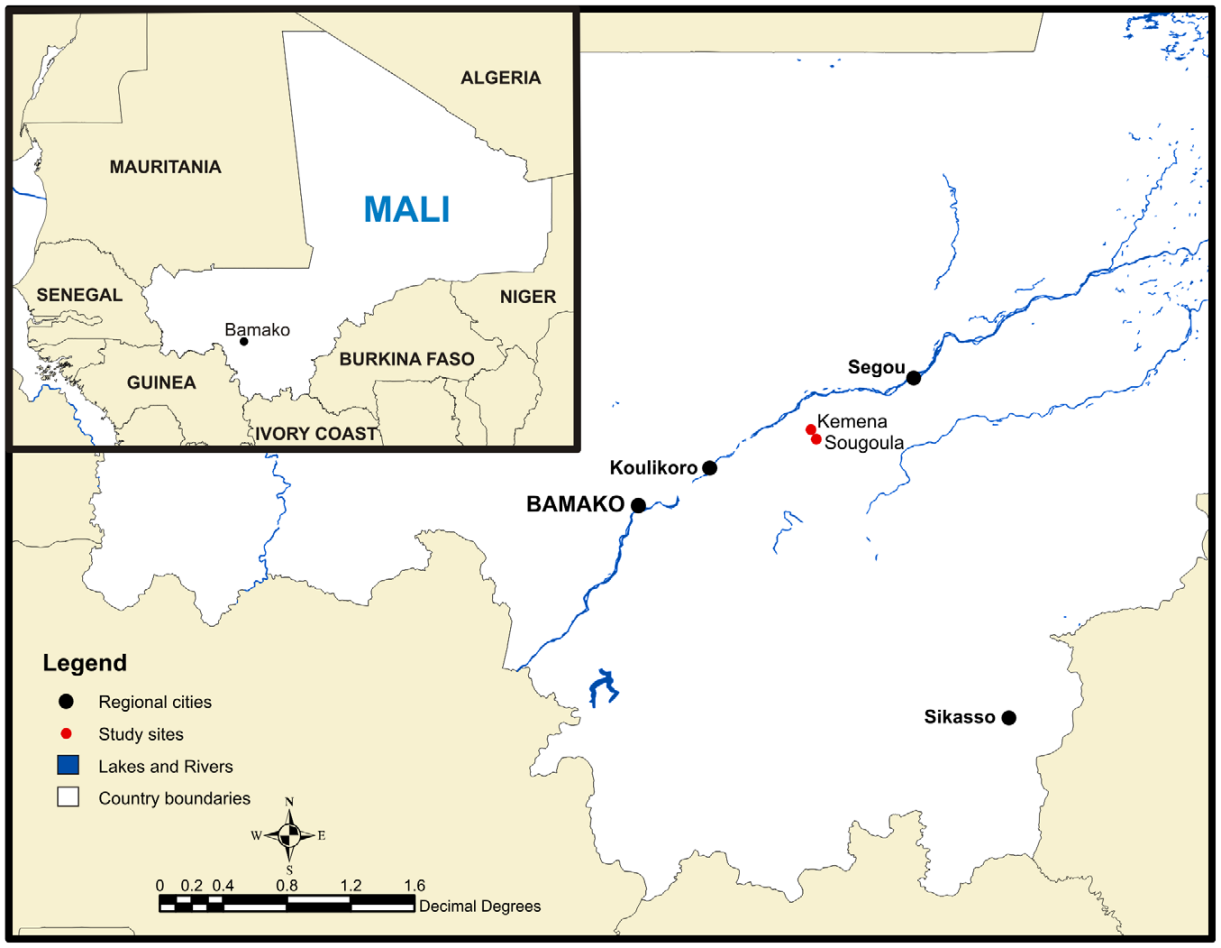
Map of Kemena and Sougoula, Region of Segou, Mali. The villages of Kemena and Sougoula are situated between the cities of Bamako and Segou. The insert illustrates the location of Mali in relation to other countries in West Africa.

The study was carried out in two villages, Kemena (13°7′30.50″N, 6°54′46.30″W) and Sougoula (13°5′24.97″N, 6°53′11.86″W), located 180 km northeast of Bamako, the capital of Mali (Figure 1). The villages are 5 km apart and share the same topography and climate. The villages are situated on a flat plain, and the natural vegetation is characterized by savannah grasses and shrubs. The climate is subtropical to arid and is hot and dry from February to June (27–34°C), mild, rainy, and humid from June to November (27–29°F), and cool and dry from November to February (25–28°C). The population consists mainly of farmers, and the basic food crops are millet, sorghum, peanuts, and peas. Cattle, goats, and sheep are maintained inside the villages. Both villages have similar multi-ethnic societies, represented by a Bambara majority and Peulh and Sarakole minorities.

### Enrollment of study subjects

Prior to the study, a census was conducted of all permanent residents in the two villages. The position of every house within each village was mapped using GPS and the houses were numbered sequentially. Local guides invited families from adjacent houses to the local school house located at the perimeter of each village to insure full coverage of the population. Families were welcomed at the school and provided with a protocol identification card comprising their name, location and a color picture. Thereafter, they received an explanation of the aims of the study and those agreeing to participate were asked to sign a consent form. Parents or guardians were asked to sign on behalf of participating children. All children younger than one year old were excluded from the study.

### Leishmanin skin test (LST)

The LST (leishmanin, LOT 124; Institute Pasteur, Tehran) was performed at the beginning of the study and at approximately 1 and 2 years later. Briefly, 0.1 ml of leishmanin was injected intradermally in the left forearm. Readings were taken 48 to 72 hours after the injection using a ball point pen to determine the size of the induration. Measurements with a diameter greater than 5 mm were considered positive [14]. Only individuals who were negative during the preceding LST survey were re-tested the following year.

### Statistical analysis

Results were analyzed using the Statistical Package for the Social Sciences (SPSS, Chicago, IL, USA). Fisher's exact test was used to assess the association between infection and demographic variables. Age means between villages were compared using an independent samples *t*-test. One-way ANOVA with Bonferroni's multiple comparison test was applied to evaluate the difference in the mean size of the LST reaction between the two study sites. Values below P*<*0.05 were considered statistically significant.

To assess geographic clustering of cases, coordinates of households were determined using a GPS receiver (Trimble, Sunnyvale, CA, USA). The coordinates were superimposed onto satellite images acquired from DigitalGlobe Incorporated (images were taken May 2006 by the QuickBird satellite). Spatial-scan statistical software (SaTScan™ v8.0 software) was used to map the location of the LST-positive individuals. The performed scans were purely spatial and accordingly were tested under the Poisson model [15]. The spatial software calculates a P value and log likelihood ratio to determine the statistical significance of any detected clusters.

## Results

### Study population

We enrolled a total of 1530 individuals, 663 from Kemena and 867 from Sougoula. The mean age of all study participants was 20 years (SD±19) and included 1- to 92-year-old individuals (Table 1). Seventy-five percent of the population was <30 years old and, nearly 53% were female. No differences in the age mean or sex distribution were found between the two villages (Table 1).

**Table 1:** Sex and age of individuals in the villages of Kemena and Sougoula at the time of enrollment.

	Kemena	Sougoula	Total	P value
Females (%)	348/663 (52.9)	461/867 (53.1)	809/1530 (52.9)	0.983
Mean age in years (SD)	21 (±19)	20 (±19)	20 (±19)	0.528

### Prevalence of *Leishmania* infection in Kemena and Sougoula

**Figure 2:**
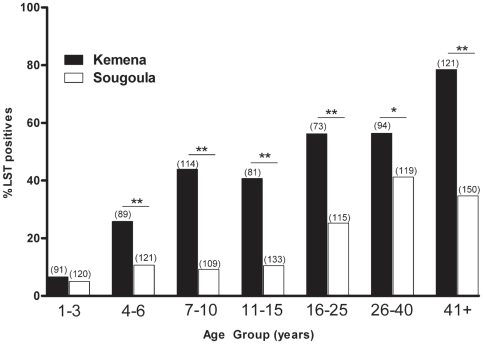
Prevalence of *Leishmania* infection by age group. Frequency of LST-positive individuals from Kemena (black bars) and Sougoula (white bars) by age group. The numbers in parenthesis represent the total number of individuals in each age group. Asterisks indicate statistical significance (* P<0.005 or ** P<0.001).

**Figure 3:**
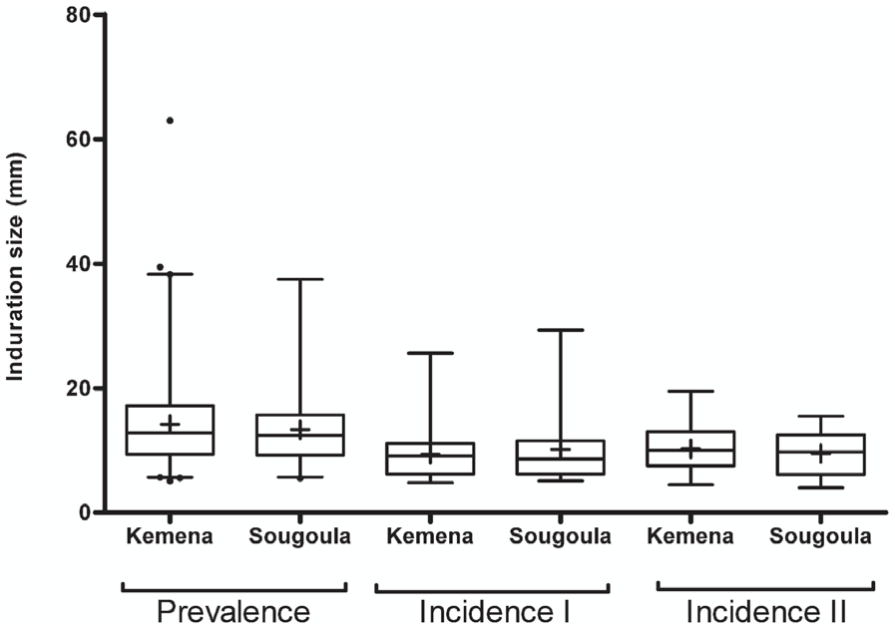
LST reaction size in Kemena and Sougoula. Induration size of LST reactions for study subjects in Kemena and Sougoula during prevalence and incidence surveys. All measurements over zero were included in the “box and whiskers” plot. The box represents the 25th and 75th percentiles. The median is represented by a solid horizontal line and the mean by a (+). The whiskers of the graph show the 1st percentile to the 99th percentile. Values lower than the first percentile and greater than the 99th percentile are represented by a (•).

In May 2006, we determined the prevalence of *Leishmania* infection by conducting a baseline LST survey of Kemena and Sougoula residents. The proportions of LST-positive (LST^+^) individuals differed by more than twofold between the villages: 45.4% in Kemena and 19.9% in Sougoula (Table 2). There was an age-associated increase in the frequency of LST^+^ individuals in both villages, although it was more pronounced in Kemena (Figure 2). Analysis of these data suggested that residents of Kemena have a more than threefold increased risk of *Leishmania* infection compared with residents of Sougoula (OR: 3.36; CI: 2.66–4.18; P<0.0005). Furthermore, the population of Kemena presented a significantly higher prevalence of infection (P<0.05) among all age groups except children less than 3 years old (Figure 2). Importantly, there was no statistical significance in the mean size of the LST reaction in inhabitants of Kemena compared to Sougoula (Figure 3).

**Table 2:** Prevalence and annual incidence of *Leishmania* infection, determined using the leishmanin skin test (LST), in individuals living in the villages of Kemena and Sougoula.

Survey	Date	Number LST positive (%)		Odds ratio	P value
		Kemena	Sougoula		
Prevalence	May 2006	301/663 (45.4)	173/867 (19.9)	3.36 (2.66–4.18)	<0.005
Incidence I	May 2007	53/287 (18.5)	32/566 (5.7)	3.78 (2.45–6.18)	<0.005
Incidence II	May 2008	32/189 (17.0)	27/470 (5.7)	3.36 (1.95–5.80)	<0.005

### Incidence of *Leishmania* infection in Kemena and Sougoula

To estimate the annual incidence of *Leishmania* infection in the two study villages, we re-tested the LST-negative (LST^−^) individuals 1 and 2 years later. The annual incidence of *Leishmania* infection in both villages combined was 9.9% (85/853) in 2007 and 8.9% (59/659) in 2008 (Table 2). In both years, we observed a higher incidence of *Leishmania* infection in Kemena than in Sougoula (18.5% vs. 5.7% in 2007 and 17.0% vs. 5.7% in 2008; P<0.001) (Table 2). Analysis of these data indicated that the risk of *Leishmania* infection was significantly higher in Kemena than in Sougoula during 2007 (OR: 3.78; CI: 2.45–6.18; P<0.005) and 2008 (OR: 3.36; CI: 1.95–5.80; P<0.005). The consistent incidence rates provide evidence that the rate of *Leishmania* transmission was stable in both villages over the 2-year study period. As was observed for prevalence, the mean size of the LST reaction was not significantly different when comparing the populations of Kemena and Sougoula for both incidence surveys (Figure 3).

### Spatial distribution of infected individuals

**Figure 4:**
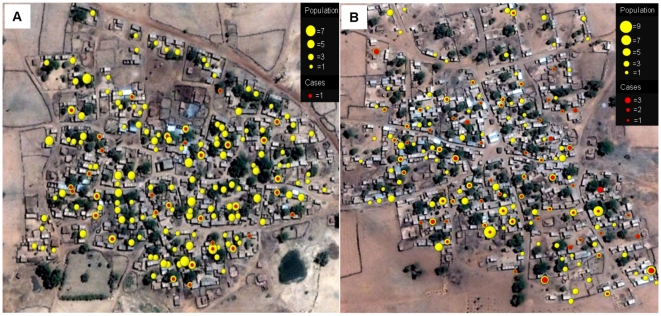
Spatial distribution of LST-positive individuals. Combined distribution of LST-positive individuals (red circles) during 2007 and 2008 relative to number of inhabitants per household (yellow circles) in the village of Kemena (A) and Sougoula (B). Diameters of yellow and red circles are proportional to the number of individuals in each location.

Kemena and Sougoula households each occupy an area of roughly 0.4 km^2^ and radiate from a central mosque in a circular pattern. Figure 4 shows a map of the number of LST^+^ individuals by household for Kemena (Figure 4A) and Sougoula (Figure 4B) for the combined incidence data of 2007 and 2008. Yellow circles represent population by household, and red circles represent LST^+^ individuals. Spatial analysis determined that there is no geographical clustering of LST^+^ individuals within either village.

## Discussion

Apart from a single publication [12], there has been no further report of CL in Mali the past 20 years. In this study, we used the LST to determine infection by *Leishmania* parasites [16]–[19]. A positive LST reaction relies on an in vivo cellular immune response specific to parasite molecules and is a measure of exposure to *Leishmania* parasites irrespective of disease presentation. Based on two annual incidence rates, our data suggest that transmission of *Leishmania* is active in the villages of Kemena and Sougoula. The high prevalence of LST^+^ individuals in these two villages was similar to prevalence data reported previously from other parts of the country [6]. This may be a reflection of the stable endemicity of *Leishmania* transmission in Mali over time. In our study villages, we have shown an increase in the number of LST^+^ individuals with age, suggesting that this is not a recent focus. The presence of LST^−^ individuals in the older age group (>41 years) may indicate a moderate force of infection. Our data show that most transmission is occurring after the age of 3 years with males and females equally affected, suggesting that transmission is not related to occupational or other gender-associated tasks.

The statistically significant difference in prevalence of infection between the two villages was unexpected, because they are located only 5 km apart and share similar geographical and human behavioral features. Sougoula was founded more than 300 years ago during the Bambara Empire [20],[21] and Kemena was established a couple of decades later. Both villages retain urbanization patterns from ancient times, and there have been no substantial differences between the two villages that could account for the increased risk of *Leishmania* infection observed in Kemena compared with Sougoula. The higher risk of infection in Kemena was maintained in two subsequent surveys, with very similar incidence rates over time. These differences may be due to a higher concentration of rodent reservoirs and, in turn, a higher infection rate of vector sand flies in Kemena. Indeed, the uniform distribution of LST^+^ individuals throughout the villages (Figure 4) may reflect peridomestic transmission, with reservoir and vector populations present either in or around houses. We have previously established the presence of *P. duboscqi*, an incriminated vector of CL in sub-Saharan Africa [23]–[26], in Kemena [27]. Additionally, examination of households in the villages revealed the presence of active rodent burrows. This may be of consequence, as rodents are the established reservoirs of CL due to *L. major* [22]. Incrimination of rodents in the village as reservoirs and finding infected sand flies inside the villages will support the hypothesis that transmission is peridomestic. Moreover, comparison of the density and infection rates of reservoir and vector populations may help elucidate the cause of the observed discrepancy in the incidence of *Leishmania* infection in the two villages. It is important to note that the mean size of the LST reaction was similar for the two villages in all the conducted surveys (Figure 3). This suggests that the observed difference in LST positivity was not the result of the non-responsiveness of either population to leishmanin.

In conclusion, the results of this study highlight the fact that there is active transmission of *Leishmania* in Kemena and Sougoula, located in Central Mali. Our findings show a significant discrepancy in CL prevalence and incidence. Studies are under way to elucidate the cause of these differences.

## Supporting Information

Checklist S1 STROBE checklist.
(0.06 MB DOC)
